# Efficacy and safety of the needle rendezvous technique for infrainguinal arterial calcified lesions

**DOI:** 10.1186/s42155-024-00490-2

**Published:** 2024-10-29

**Authors:** Takuya Haraguchi, Masanaga Tsujimoto, Yoshifumi Kashima, Yuhei Kasai, Katsuhiko Sato, Tsutomu Fujita

**Affiliations:** Department of Cardiology, Sapporo Heart Center, Asia Medical Group, Sapporo Cardio Vascular Clinic, North 49, East 16, 8-1, Higashi Ward, Sapporo, Hokkaido 007-0849 Japan

**Keywords:** Chronic total occlusion, Calcification, Needle, Peripheral artery disease, Chronic limb-threatening ischemia

## Abstract

**Background:**

Lower extremity artery disease is increasingly prevalent, and complex lesions such as calcified chronic total occlusions pose significant challenges during endovascular therapy. The needle rendezvous technique, which involves puncturing a needle toward the guidewire within the lesion or lumen and advancing the guidewire into the needle lumen to achieve guidewire externalization, offers a potential solution. If device passage remains challenging, the Rendezvous-PIERCE technique can be subsequently employed by advancing the needle over the externalized guidewire to modify the lesion directly. This study aimed to evaluate the procedural outcomes of needle rendezvous in infrainguinal arterial occlusive lesions.

**Methods:**

This single-center, retrospective, single-arm study included patients treated with needle rendezvous between August 2020 and March 2024. The primary outcome was technical success rate, defined as the device passage following guidewire externalization using needle rendezvous. Secondary outcomes included the rates of procedural success, complications, and 30-day clinical-driven target lesion revascularization (CDTLR).

**Results:**

Twenty-five patients (25 limbs) with 52% on hemodialysis and 80% having chronic limb-threatening ischemia in 52% and 80% were enrolled. All cases involved bilateral calcified occlusions, and 72% targeted the infrapopliteal artery segment. The average needle rendezvous time was 3.7 ± 2.0 min. Rendezvous-PIERCE was performed in 28% of cases. All cases achieved 100% technical and procedural success, with no procedure-related complications. The 30-day CDTLR rate was 8%, limited to below-the-knee lesions.

**Conclusions:**

Needle rendezvous is a safe and effective technique for treating complex infrainguinal arterial occlusions, providing a viable alternative when conventional methods fail.

**Supplementary Information:**

The online version contains supplementary material available at 10.1186/s42155-024-00490-2.

## Background

Owing to the increasing incidence of lower extremity artery disease (LEAD), the prevalence of endovascular therapy (EVT) has increased [1]. Advances in medical devices have significantly improved clinical and procedural outcomes [2]. However, patients with LEAD frequently present with suboptimal patient and lesion characteristics, such as diabetes mellitus and chronic kidney disease, leading to calcified lesions and chronic total occlusions (CTOs) [3]. These complex lesions obstruct the antegrade passage of devices, such as guidewires, microcatheters, balloons, and stents. In difficult-to-cross lesions, a retrograde approach is commonly performed to achieve guidewire externalization, facilitating the passage of microcatheters and balloons [4].

In the retrograde approach, the needle punctures the distal lumen in a retrograde manner, allowing the insertion of a guidewire and a microcatheter or sheath for retrograde wiring [5]. However, if the targeted distal lumen is small, severely calcified, or has a short margin between the puncture site and the distal cap of the occlusion, the microcatheter and sheath may not be successfully inserted even if the needle punctures the lumen and a guidewire is introduced. Consequently, the retrograde approach may fail, resulting in overall procedural failure. Furthermore, severely calcified lesions may inhibit the penetration of conventional re-entry devices and the success of percutaneous direct needle puncture of the calcified plaque (PIERCE) technique, which creates cracks in the calcification to facilitate the device passage by direct external puncture using a needle [6].

**Fig. 1:**
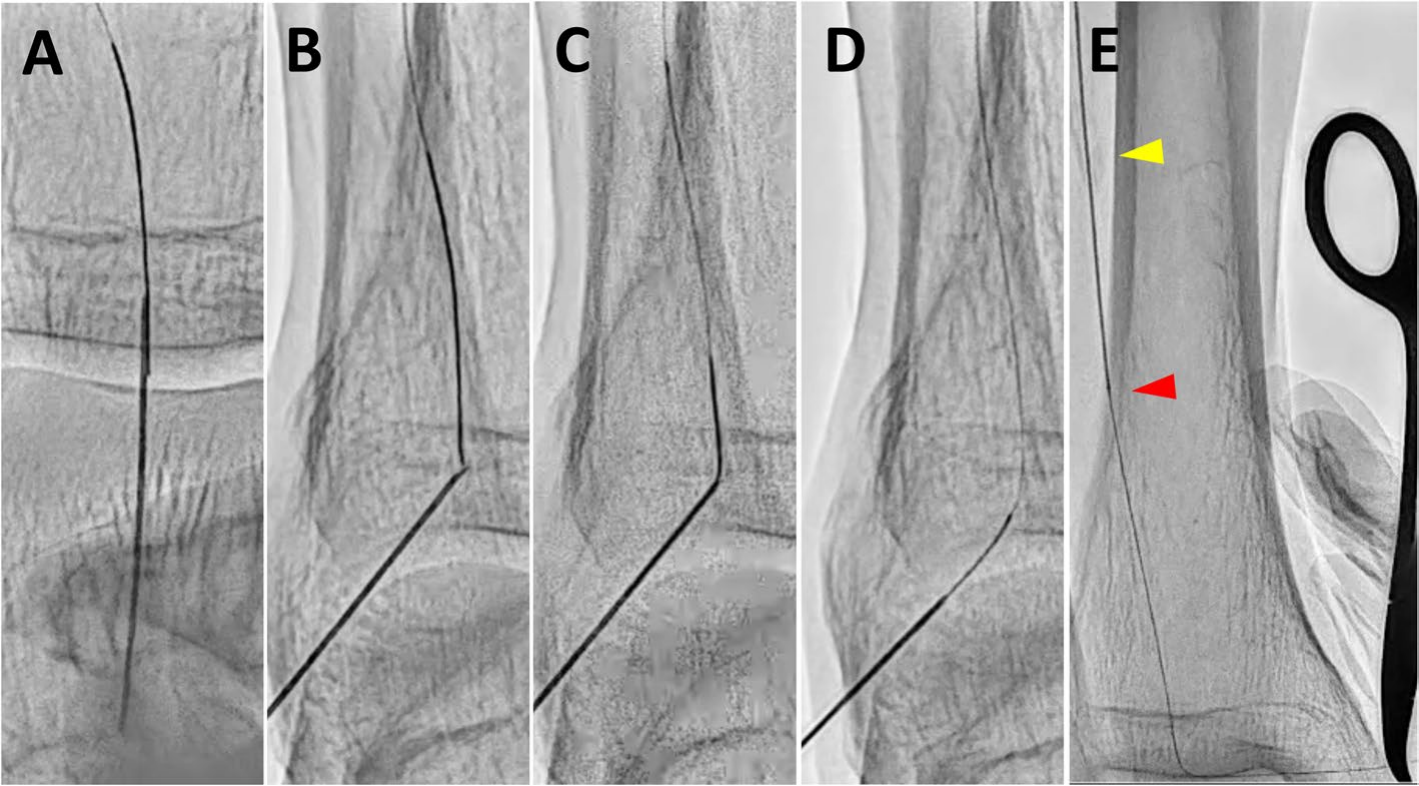
Procedural process of needle rendezvous. **A** The needle is aligned with the target guidewire and inserted toward it. **B** The depth of the needle is adjusted to match the position of the guidewire. **C** The guidewire is advanced into the needle lumen. **D** The needle is removed to externalize the guidewire. **E** The externalized guidewire is held with forceps, and the antegrade microcatheter (red arrow) is advanced across the uncrossable lesion (yellow arrow) to create a hole in the lesion. *Print color requested*

**Fig. 2:**
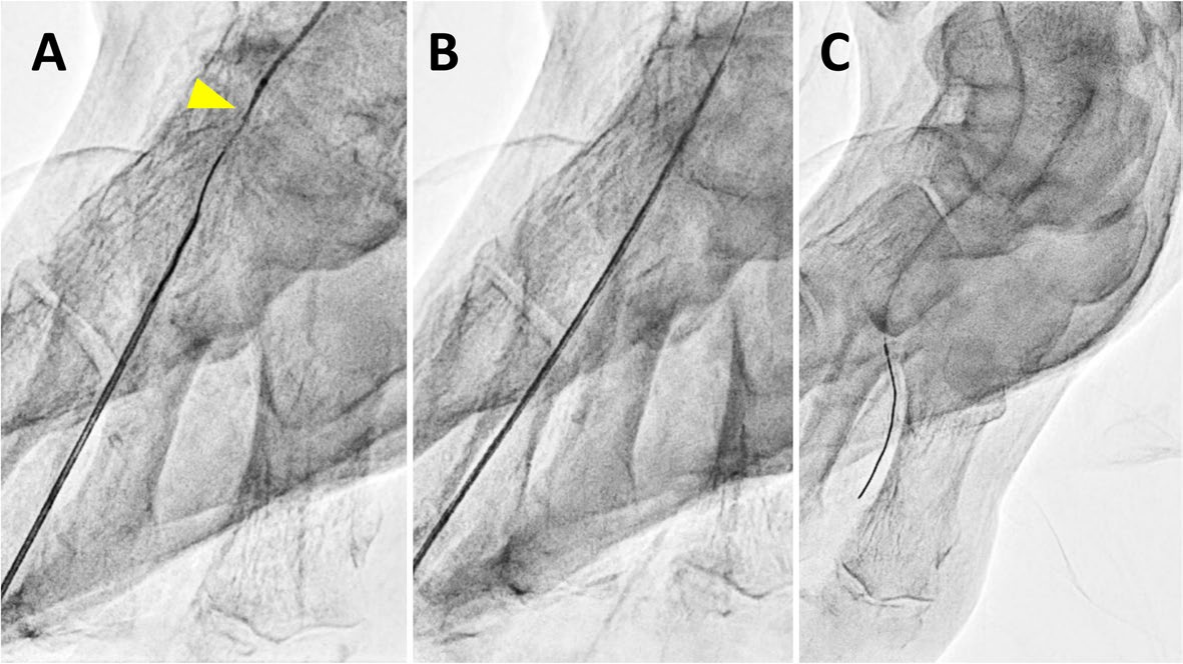
Procedural process of Rendezvous-PIERCE. **A **Following needle rendezvous, attempts to cross the difficult-to-cross lesion (yellow arrow) with a balloon and a microcatheter are unsuccessful. **B** The needle is then inserted over the externalized guidewire from the puncture site into the vessel, successfully crossing the lesion and creating a needle hole within the lesion. **C** A balloon is subsequently advanced and used to dilate the lesion. *Print color requested*

**Fig. 3:**
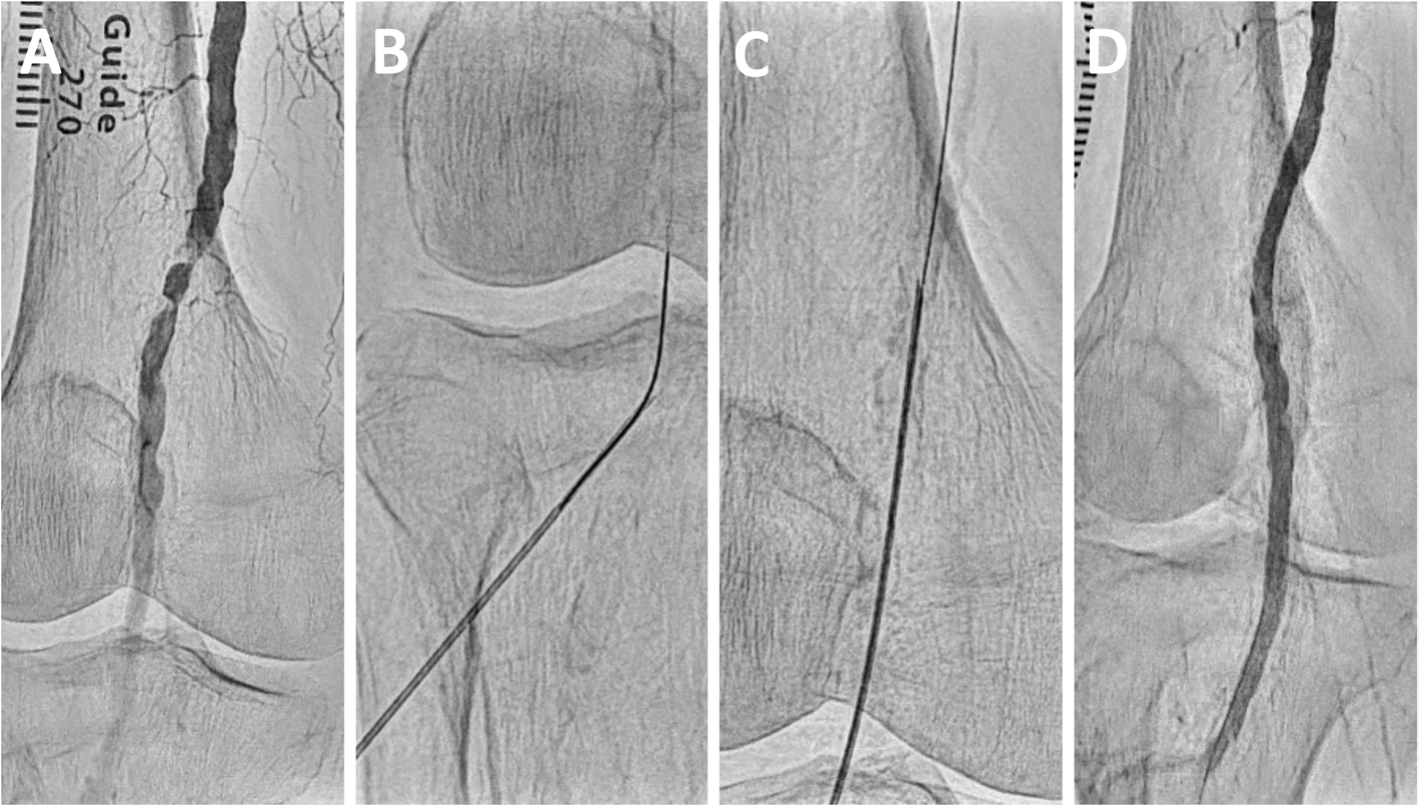
Rendezvous-PIERCE for the severely calcified lesion in the popliteal artery. **A **The initial angiogram demonstrated an eccentric calcified lesion at the proximal segment of the popliteal artery. **B** Needle rendezvous was performed using a 20-gauge, 20 cm needle to achieve guidewire externalization, as no device was able to cross the lesion. **C** The needle was inserted from the puncture site, and the lesion was crossed to modify its intimal calcification. **D** The final angiogram demonstrated sufficient flow after successful crossing and full dilation with a 5.0 mm balloon

Previous reports have demonstrated some needle-based treatment methods to address these issues, achieving procedural success in cases of complex occlusive lesions [7–12]. Based on these studies, the challenges posed by conventional methods can be effectively addressed using the needle rendezvous [7]. This approach entails puncturing a needle towards the guidewire within the lesion or lumen, then advancing the guidewire into the needle lumen to achieve the guidewire externalization, allowing for the passage of devices through the externalized guidewire (Fig. 1A–E and Supplementary video 1). If device passage remains difficult despite performing the needle rendezvous, the needle is advanced over the externalized guidewire using wire tension, inserted into the vessel from the puncture site, and passed across the lesion to directly modify the uncrossable lesion, referred to as Rendezvous-PIERCE (Fig. 2A–C and Supplementary video 2; Fig. 3A–D and Supplementary video 3) [8]. In previous case reports, the needle rendezvous was applied for re-entry in cases where conventional devices had proven difficult, and revascularization was successfully performed using the pave-and-crack technique with Viabahn (W.L., Gore & Associates, Inc., USA) implantation [7, 9]. Needle rendezvous has been reported as a helpful technique for treating difficult-to-cross lesions; however, clinical data involving this technique are limited. We aimed to evaluate the procedural outcomes of needle rendezvous in peripheral interventions for infrainguinal arterial occlusive lesions in a real-world clinical setting.

## Methods

### Study design and patient population

This single-center, retrospective, single-arm study was conducted at the Sapporo Heart Center in Japan. Between August 2020 and March 2024, 2,187 patients with symptomatic LEAD underwent EVT. From this cohort, 30 patients (30 limbs) who underwent the needle rendezvous procedure in the infrainguinal artery and were classified as having a Rutherford classification of 2–6 were selectively enrolled. Patients who had undergone the needle rendezvous technique primarily for sheath insertion were excluded. The study protocol was approved by the Ethics Committee of the Sapporo Heart Center and adhered to the principles of the Declaration of Helsinki. The requirement for written informed consent was waived because of the retrospective design of the study.

### Endovascular procedures and medical therapy

Patients with symptomatic femoropopliteal and below-the-knee arterial occlusive lesions and a Rutherford classification of 2–6 were identified as candidates for EVT. The common femoral artery on the approach side of the target lesion was accessed using a 5- or 6-Fr sheath. An initial dose of 5000 IU of unfractionated heparin was administered, with additional doses to achieve an activated coagulation time of at least 200 s. The lesions were crossed using 0.014- and 0.018-inch guidewires supported by a workhorse microcatheter. The needle rendezvous technique was applied if the microcatheter and balloon had difficulty crossing the lesions over the guidewire or if a conventional re-entry device could not penetrate the wall of the targeted artery. Depending on the vessel diameter and lesion location, an 18-gauge (64 mm, Terumo Corporation, Japan), a 20-gauge (105–200 mm, Medikit Co., Ltd., Japan), or a 22-gauge (42 mm, Medikit Co., Ltd., Japan) single wall hollow needle was used for needle rendezvous. The size and type of microcatheter and balloon used were selected based on the lesion morphology and location under angiographic guidance. If the conventional device could not cross the lesion, even in the case of guidewire externalization by needle rendezvous, the Rendezvous-PIERCE technique was applied [8]. If balloon dilatation was not fully achieved, the Rendezvous-PIERCE technique was repeated to modify the lesion. Scaffold implantation and drug-coated balloon angioplasty were performed on femoropopliteal and infrapopliteal lesions after plain old balloon angioplasty at the operator’s discretion. The pave-and-crack technique using Viabahn was employed in cases of diffuse calcified occlusion, in which suboptimal procedure outcomes were expected after the standard strategy [7, 9, 13]. Atherectomy devices were unavailable for below-the-knee lesions. Hemostasis of the needle rendezvous puncture site was achieved using balloon tamponade and compression with a bandage.

After the procedure, dual antiplatelet therapy (100 mg of aspirin and 75 mg of clopidogrel daily) was administered. Patients receiving anticoagulant therapy received a combination of anticoagulant and dual antiplatelet therapy for 1 month, followed by long-term anticoagulant and aspirin therapy.

### Study outcomes and follow-up

The primary outcome was technical success, defined as the successful passage of the device after guidewire externalization using the needle rendezvous technique. The secondary outcomes included procedural success (residual stenosis < 30% without flow-limiting antegrade flow and the absence of procedural complications), procedure-related complications, and early clinical-driven target lesion revascularization (CDTLR; repeat EVT or surgical bypass for lesions exhibiting recurrent symptoms and restenosis within 30 days of the initial procedure). Restenosis was identified by having either a peak systolic velocity ratio greater than 2.4 on duplex ultrasonography or over 50% stenosis, as evident in follow-up computed tomography angiograms [14, 15] The severity of LEAD was categorized according to the Rutherford classification: mild-to-severe intermittent claudication (classes 1–3) and CLTI with and without tissue loss (classes 4–6) [16]. Calcification laterality was assessed using the Peripheral Artery Calcification Scoring System (PACSS) classification [17].

### Statistical analysis

Continuous variables are presented as the means ± standard deviations and were compared using the unpaired *t*-test or the Mann–Whitney *U* test. Categorical variables are expressed as numbers (percentages) and were compared using the chi-square test. Significance was set at *P* < 0.050. Statistical analyses were performed using IBM SPSS Statistics software (version 29.0).

## Results

### Baseline clinical characteristics

Needle rendezvous was performed on 30 limbs of 30 patients; however, five patients were excluded from this study because needle rendezvous was used for sheath insertion. The patients’ baseline characteristics are presented in Table 1. The mean age of the patients was 77.0 ± 8.3 years, and 17 patients (68%) were men. The proportions of patients receiving ambulatory care, those with chronic kidney disease, and those who underwent hemodialysis were 52%, 80%, and 52%, respectively. Patients with CLTI accounted for 80% of the study cohort. Antiplatelet and anticoagulant drugs were administered to 88% and 40% of patients, respectively. All lesions were characterized by CTOs and classified as PACSS grade 4 for calcifications. The target lesions were located in the femoropopliteal and below-the-knee arteries in 56% and 80% of patients, respectively. The mean operative time was 187 ± 93 min. The total amount of heparin administered was 6600 ± 1258 IU.

**Table 1:** Baseline patient and lesion characteristics

	Needle rendezvous *n* = 25
Age, years	77.0 ± 8.3
Male sex	17 (68%)
Body mass index, kg/m^2^	21.3 ± 2.9
Ambulatory/ wheel chair/ bedridden	13 (52%)/ 9 (36%)/ 3 (12%)
Diabetes mellitus	17 (68%)
Chronic kidney disease/ hemodialysis	20 (80%)/ 13 (52%)
Stroke/ coronary artery disease	7 (28%)/ 17 (68%)
Antiplatelet/ anticoagulant drug	22 (88%)/ 10 (40%)
Statin	13 (52%)
Chronic limb-threatening ischemia	20 (80%)
Femoropopliteal/below-the-knee lesions	14 (56%)/ 20 (80%)
Distal reference vessel diameter, mm	3.0 ± 1.0 (1.3–5.3)
Chronic total occlusion	25 (100%)
Calcification, graded as PACSS grade 4	25 (100%)
Below-the-knee poor-runoff ≤1	19 (76%)
Intravascular ultrasound use	7 (28%)
Scaffold/drug-coated balloon use	5 (20%)/ 1 (4%)
Heparin dose, mL	6600 ± 1258
Operative time, min	187 ± 93
Contrast dose, mL	175 ± 103

Data are presented as number (percentage) or mean ± standard deviation (range) unless otherwise specified

*PACSS* Peripheral Artery Calcium Scoring System

### Technical characteristics and study outcomes

Table 2 presents the baseline needle rendezvous characteristics. The target sites for needle rendezvous were the superficial femoral, below-the-knee, and below-the-ankle arteries in 28%, 40%, and 32% of patients, respectively. The puncture vessel diameter was 2.8 ± 1.5 mm (range, 1.1–6.8 mm). The needle gauges used in this technique were 18, 20, and 22-gauge in 40%, 36%, and 24% of the cases, respectively. The diameter of the guidewire used for needle rendezvous was 0.014 inches. The mean tip load was 17 ± 28 g (median, 3 g; range, 1–100 g). Guidewires with soft, moderate, and hard tip loads were used in 52%, 32%, and 16% of the cases, respectively. Tapered guidewires accounted for 24% of the guidewires used, and plastic- and hydrophilic-coated guidewires were used in 52% and 48% of the cases, respectively. The goals of needle rendezvous were device delivery in 64% of the cases (36% for microcatheters and 28% for balloons) and a procedure strategy in 56% of the cases (28% for Rendezvous-PIERCE, 28% for re-entry for difficult-to-cross CTO. The procedural time for needle rendezvous was 3.7 ± 2.0 min (range, 1–9 min). Table 3 presents the study outcomes. The technical and procedural success rates were 100%. No procedure-related complications occurred. The early CDTLR rate was 8% (*n* = 2/25) and was performed exclusively in below-the-knee artery lesions.

**Table 2:** Baseline needle rendezvous characteristics

Characteristic	Needle rendezvous *n* = 25
Needle rendezvous time, min	3.7 ± 2.0 (1–9)
Needle rendezvous site	
Superficial femoral artery	7 (28%)
Below-the-knee artery	10 (40%)
Below-the-ankle artery	8 (32%)
Puncture vessel diameter, mm	2.8 ± 1.5 (1.1–6.8)
Needle use, 18/ 20/ 22 gauge	10 (40%)/ 9 (36%)/ 6 (24%)
Rendezvous guidewire	
0.014-inch/ 0.018-inch	23 (92%)/ 2(8%)
Tip load (g)	17 ± 28 [3] (1–100)
Soft/ moderate/ hard type	13 (52%)/ 8 (32%)/ 4 (16%)
Tapered/ non-tapered type	6 (24%)/ 19 (76%)
Plastic coat/ hydrophilic coat	13 (52%)/ 12 (48%)
Purpose	
Delivery; microcatheter/ balloon	9 (36%)/ 7 (28%)
Procedure; Rendezvous-PIERCE/ Re-entry	7 (28%)/ 7 (28%)

Data are presented as number (percentage) or mean ± standard deviation [median] (range) unless otherwise specified

**Table 3:** Study outcomes

	Needle rendezvous *n* = 25
Needle rendezvous technical success	25 (100%)
Procedure success	25 (100%)
Complication	0 (0%)
Vessel rupture/ guidewire cut/ puncture site trouble	0 (0%)/ 0 (0%)/ 0 (0%)
Early target lesion revascularization	2 (8%)

Data are presented as number (percentage)

The comparison between the Needle rendezvous and Rendezvous-PIERCE groups is presented in Supplementary Table A. Patients in the Needle rendezvous group were older (79.2 ± 7.5 vs. 71.3 ± 8.2 years, *p* = 0.025) and had a lower prevalence of diabetes mellitus (56% vs. 100%, *p* = 0.040). The total procedure time in the Rendezvous-PIERCE group was numerically shorter but not statistically significant (192 ± 100 vs. 173 ± 76 min, *p* = 0.334). Both groups achieved 100% technical and procedural success with no complications. Additionally, there was no significant difference in the rate of early CDTLR between the groups (6% vs. 14%, *p* = 0.491).

## Discussion

In the present study, we assessed the feasibility and safety of the needle rendezvous technique for guidewire externalization in cases where the conventional approach failed. The technical and procedural success rates of needle rendezvous were 100%. No procedure-related complications occurred. The early CDTLR rate was 8% in only below-the-knee artery lesions.

Recent advances in medical devices have improved procedural success rates; however, not all cases resulted in successful outcomes [18]. Clinical outcomes are inferior in patients with failure to cross lesions [19]. Guidewire externalization using a bidirectional approach facilitates successful device delivery through uncrossable lesions [20]. A sheath or microcatheter should be retrogradely inserted through the distal vessel for the retrograde approach. Needle rendezvous can provide a valuable alternative if the target distal vessel is unsuitable for a retrograde approach. Although needle rendezvous may appear challenging due to the requirement of inserting a guidewire into the needle lumen, it is feasible because the lumen diameter of 18- or 20-gauge needles is larger than the outer diameter of microcatheters compatible with 0.014- and 0.018-inch guidewires. Therefore, when guidewire rendezvous is feasible, needle rendezvous can be successfully performed [21]. Consequently, the average procedure time for needle rendezvous in the present study was relatively shorter than expected, at 3.7 min. 0.014-inch guidewires were predominantly used, although 0.018-inch guidewires were also employed. Thinner guidewires are generally preferred for this technique. Additionally, soft, moderately tip-loaded, and non-tapered guidewires were primarily used. Plastic- or hydrophilic-coated guidewires were also utilized, resulting in less friction between the needle lumen and the guidewire during advancement. Coated guidewires are preferable over uncoated ones due to their lower risk of guidewire-related complications, such as guidewire cutting. No guidewire-related complications were observed in this study. Antiplatelets (88%), anticoagulants (28%), and heparin (6600 IU) were administered; however, no puncture site complications, such as bleeding or vessel rupture, were noted. The size of the puncture hole corresponded to the outer diameter of the needle used (approximately 0.9 mm for a 20-gauge needle), allowing hemostasis to be achieved with external pressure alone, provided the target artery was not deep. This procedure was primarily performed in the infrapopliteal arterial segment, which may have contributed to the absence of complications. Experienced interventionists performed the needle rendezvous technique and achieved hemostasis both externally and internally using a balloon and bandage, which likely accounts for the lack of procedure-related complications.

The use of a re-entry device (Outback, Cordis, FL, USA) and catheter (Wingman, Reflow Medical, CA, USA) with a needle at the tip to facilitate procedural success in cases where device passage is difficult has been previously reported [22, 23]. However, these devices were unable to penetrate all calcified lesions. In such cases, previous reports have described the effectiveness of extraluminal and intraluminal approaches, known as the PIERCE and inner PIERCE techniques, which fracture calcification with a needle [6, 24]. The PIERCE technique, as an extraluminal needle method, is designed to modify deep calcification rather than superficial calcification. In contrast, the inner PIERCE technique targets superficial calcification. The inner PIERCE technique has been studied exclusively in infrapopliteal arterial lesions, with a reported lesion crossing rate of 100%, a procedural success rate of 94%, and no complications [24]. In the present study, Rendezvous-PIERCE, a combination of needle rendezvous and inner PIERCE, was employed when the retrograde approach using needle rendezvous proved challenging, achieving a 100% procedural success rate with no complications. The procedure time for Rendezvous-PIERCE following Needle Rendezvous, although not statistically significant, was numerically shorter than that of Needle Rendezvous alone, indicating the potential for reducing the overall procedure time. This suggests that modifying calcification with a needle followed by balloon angioplasty may be more efficient than using a microcatheter for the dotter effect or attempting to cross and dilate with a balloon alone. Furthermore, since all patients in the present study had CTOs with PACSS grade 4 and complex lesions, the pave-and-crack technique using needle rendezvous (needle bypass and re-entry technique) was employed to treat severely diffuse calcified lesions that could not be successfully addressed with conventional devices [7, 9]. Notably, this novel, operator-developed procedure utilizes a needle for crossing complex lesions; however, it is both cost-effective and practical compared to conventional devices. Needle-based techniques can be applied in challenging cases. Given the small sample size in this study, further research is warranted to generalize this technique.

### Study limitations

First, this study’s retrospective, nonrandomized, single-center, single-arm observational design may have led to case selection bias and nonrandom assignment. Second, the small sample size may have affected the reliability of our results. Third, the needle rendezvous technique was performed in only 1.3% (*n* = 30/2187) of the patients during the study period. This technique helps achieve procedural success in difficult-to-cross lesions; however, further studies are needed to evaluate its normalization for treating complex lesions. Fourth, only cases of CTO lesions with severe calcification, classified as PACSS grade 4 lesions, were included in this study. Therefore, the procedural outcomes of less severely calcified occlusions are unclear. Finally, this technique can be performed on the infrainguinal arteries but not on suprainguinal arteries. The learning curve involves puncturing the lumen using a needle and manipulating the guidewire to advance into the needle lumen.

## Conclusion

The needle rendezvous technique is beneficial for patients with difficult-to-cross lesions. Further large-scale clinical studies are needed to standardize this technique.

## Supplementary Information

Supplementary Material 1. Supplementary Table A: Comparison of characteristics and outcomes between needle rendezvous and Rendezvous-PIERCE.

Supplementary Material 2.  Supplementary video 1: Needle rendezvous for the dorsalis pedis artery occlusion.

Supplementary Material 3.  Supplementary video 2: Rendezvous-PIERCE for severe calcified lesion in the dorsalis pedis artery.

Supplementary Material 4.  Supplementary video 3: Rendezvous-PIERCE for severe calcified lesion in the popliteal artery.

## Data Availability

The data associated with this research are available from the corresponding author upon reasonable request.

## Abbreviations

CLTI: Chronic limb-threatening ischemia
CTO: Chronic total occlusion
EVT: Endovascular treatment
LEAD: Lower extremity artery disease
PACSS: Peripheral Artery Calcification Scoring System
TLR: Target lesion revascularization
