# Supplementary material for: Efficacy and safety of the needle rendezvous technique for infrainguinal arterial calcified lesions
Source: CVIR Endovasc. 2024 Oct 29;7:77. doi: 10.1186/s42155-024-00490-2 (PMC11522230; doi:10.1186/s42155-024-00490-2)
Supplement: Supplementary file 1 — Supplementary Material 1. Supplementary Table A: Comparison of characteristics and outcomes between needle rendezvous and Rendezvous-PIERCE. [file 42155_2024_490_MOESM1_ESM.docx]

**Supplementary Table A. Comparison of characteristics and outcomes between needle rendezvous and Rendezvous-PIERCE**

|  | Needle rendezvous  n=18 | Rendezvous-PIERCE  n=7 | P-value |
| --- | --- | --- | --- |
| Patient and lesion characteristics | | | |
| Age, years | 79.2±7.5 | 71.3±8.2 | 0.025 |
| Male sex | 11 (61%) | 6 (86%) | 0.246 |
| Body mass index, kg/m^2^ | 21.1±3.2 | 21.7±2.0 | 0.354 |
| Ambulatory/ Wheel Chair/ Bed ridden | 10 (56%)/ 7 (39%)/ 1 (6%) | 3 (43%)/ 2 (29%)/ 2 (29%) | 0.450/ 0.501/ 0.180 |
| Diabetes mellitus | 10 (56%) | 7 (100%) | 0.040 |
| Chronic kidney disease/ Hemodialysis | 14 (78%)/ 8(44%) | 6 (86%)/ 5 (71%) | 0.564/ 0.223 |
| Stroke/ Coronary artery disease | 6 (33%)/ 13 (72%) | 1 (14%)/ 4 (57%) | 0.337/ 0.393 |
| Antiplatelet/ Anticoagulant drug | 15 (83%)/ 9 (50%) | 5 (71%)/ 1 (14%) | 0.436/ 0.118 |
| Statin | 8 (44%) | 5 (71%) | 0.223 |
| Chronic limb-threatening ischemia | 13 (72%) | 7 (100%) | 0.161 |
| Femoropopliteal/ Below-the-knee lesions | 10 (56%)/ 13 (72%) | 4 (57%)/ 7 (100%) | 0.649/ 0.161 |
| Distal reference vessel diameter, mm | 3.1±1.2 | 2.8±0.5 | 0.791 |
| Chronic total occlusion | 18 (100%) | 7 (100%) | - |
| Calcification graded by the PACSS grade 4 | 18 (100%) | 7 (100%) | - |
| Below-the-knee poor-runoff ≤1 | 14 (77%) | 5 (71%) | 0.751 |
| Procedure characteristics | | | |
| Intravascular ultrasound use | 10 (56%) | 6 (86%) | 0.174 |
| Scaffold/ Drug-coated balloon use | 5 (28%)/ 0 (0%) | 0 (0%)/ 1 (14%) | 0.161/ 0.280 |
| Heparin dose, ml | 6667±1372 | 6429±976 | 0.340 |
| Operation time, min | 192±100 | 173±76 | 0.334 |
| Contrast dose, ml | 190±114 | 137±58 | 0.260 |
| Needle rendezvous time, min | 3.9±2.3 (1–9) | 3.0±0.6 (2–4) | 0.122 |
| Needle rendezvous site |  |  |  |
| Femoropopliteal artery | 6 (33%) | 2 (29%) | 0.607 |
| Below-the-knee artery | 6 (33%) | 4 (57%) | 0.261 |
| Below-the-ankle artery | 6 (33%) | 1 (14%) | 0.337 |
| Puncture vessel diameter, mm | 2.9±1.7 (1.1–6.8) | 2.6±0.7 (1.6–5.2) | 0.395 |
| Needle use, 18/ 20/ 22 gauge | 10 (56%)/ 4 (22%)/ 4 (22%) | 0 (0%)/ 5 (71%)/ 2 (29%) | 0.013/ 0.034/ 0.557 |
| Rendezvous Guidewire |  |  |  |
| 0.014-inch/ 0.018-inch | 16 (89%)/ 2 (11%) | 7 (100%)/ 0 (0%) | 0.510 |
| tip load (g) | 11±15 [3] (1–100) | 19±32 [5] (3–45) | 0.523 |
| soft/ moderate/ hard type | 9 (50%)/ 6 (33%)/ 3 (17%) | 4 (57%)/ 2 (29%)/ 1 (14%) | 0.550/ 0.607/ 0.693 |
| tapered/ non-tapered type | 5 (28%)/ 13 (72%) | 1 (14%)/ 6 (86%) | 0.443 |
| plastic coat/ hydrophilic coat | 9 (50%)/ 9 (50%) | 4 (57%)/ 3 (43%) | 0.550 |
| Study outcomes |  |  |  |
| Needle rendezvous technical success | 18 (100%) | 7 (100%) | - |
| Procedure success | 18 (100%) | 7 (100%) | - |
| Complication | 0 (0%) | 0 (0%) | - |
| vessel rupture/ guidewire cut/ puncture site trouble | 0 (0%)/ 0 (0%)/ 0 (0%) | 0 (0%)/ 0 (0%)/ 0 (0%) | - |
| Early target lesion revascularization | 1 (6%) | 1 (14%) | 0.491 |
| Data are presented as number (percentage) or mean±standard deviation unless otherwise specified.  PACSS, Peripheral Artery Calcium Scoring System | | | |
